# Boosting Autobiographical Memory and the Sense of Identity of Alzheimer Patients Through Repeated Reminiscence Workshops?

**DOI:** 10.3389/fpsyg.2021.636028

**Published:** 2021-02-15

**Authors:** Hervé Platel, Marie-Loup Eustache, Renaud Coppalle, Armelle Viard, Francis Eustache, Mathilde Groussard, Béatrice Desgranges

**Affiliations:** Normandie Univ, UNICAEN, EPHE, INSERM, U1077, CHU de Caen, Neuropsychologie et Imagerie de la Mémoire Humaine, Caen, France

**Keywords:** Alzheimer, autobiographical memory, reminiscence, sense of identity, music

## Abstract

Despite severe amnesia, some studies showed that Alzheimer Disease (AD) patients with moderate to severe dementia keep a consistent, but impoverished representation of themselves, showing preservation of the sense of identity even at severe stages of the illness. Some studies suggest that listening to music can facilitate the reminiscence of autobiographical memories and that stimulating autobiographical memory would be relevant to support the self of these patients. Consequently, we hypothesized that repeated participation to reminiscence workshops, using excerpts of familiar songs as prompts would participate to the enrichment of autobiographical memories, self-representation and sense of identity. We included a group of 20 AD patients with severe dementia residing in nursing homes. Their performances were compared to a control group of 20 matched (age, education, mood) healthy residents living in the same institutions. The experiment was conducted in three phases over a 2-week period. On phase 1, an individual assessment of sense of identity was proposed to each participant. On phase 2, participants joined musical reminiscence workshops (six sessions over 2 weeks for AD patients and 3 sessions over a week for controls). During the third phase (12 days after the first assessment), individual evaluation of autobiographical memory and a second assessment of sense of identity were proposed. Our results showed that, despite their massive amnesia syndrome, autobiographical memories of AD reached at the end of the 2 weeks the number and quality of those of matched controls. Moreover, we confirmed a continuity of self-representation in AD patients with a stable profile of the answers between the first and second individual assessments of sense of identity. However, the increase in number and episodic quality of autobiographical memories was not accompanied by an enrichment of the sense of identity. In a complementary study, new patients participated in the same paradigm, but using movie extracts as prompts, and showed very similar effects. We discuss all of these results with regard to the literature showing the significant impact of repetition on the reactivation of memory traces even in very amnestic AD patients at severe stages of the disease.

## Introduction

In psychology, the self is viewed as a complex multi-dimensional construct with a structural level of self-representation in memory (Kihlstrom et al., 2002) and a set of self-related functional processes required to evoke self-representations (Stuss et al., 2001; Morin, 2006). Self-representations, which give rise to the sense of identity, consist of personal information, stored in either semantic or episodic autobiographical memory (Klein, 2010). If autobiographical memory is indeed crucial to the construction of the sense of identity and the feeling of self-continuity (Conway, 2005), what happens to the sense of identity in patients suffering from severe episodic memory disorders and who are thus unable to re-experience personal events with “warmth and intimacy”?

Alzheimer's disease (AD) is characterized by a severe episodic memory disorder from the onset of the disease, as well as semantic memory disturbances that can become significant by the time the patient has reached the moderate stage. Indeed, it is legitimate to wonder whether residual semantic memory is sufficient to ensure the persistence of the sense of identity in these patients. Ben Malek et al. (2018) aimed to investigate the integrative meaning of self-defining memories (SDM, i.e., memories of highly significant personal events) in early stages of AD. Their results showed that, when compared to the control group, AD patients less frequently extracted meaning from their SDM and the meaning was less frequently tied to the self. El Haj and Allain (2020) showed that, although AD participants produced fewer specific SDM than controls, both populations had similar levels of emotional valence, integration, and self-continuity of these memories, at least for AD participants in the mild stages of the disease. Our previous study (Eustache et al., 2013) aimed at showing preservation of the sense of identity in AD patients at a moderate to severe stage of the illness: assessing twice their sense of identity within a 15 days interval, patients were able to introduce themselves in a very similar way, while they did not recall any circumstance and context of these assessments. In this latter study, we found a consistent self-representation among AD patients at moderate to severe stages of the disease. However, we also observed a poverty of AD responses, frequently word for word matching in both testing sessions, while the answers were more detailed for the control participants. Moreover, our results fitted with the existence of two kinds of sense of identity, as proposed by Ricoeur (1990): one using a personal semantic memory marked by sameness of the person (i.e., “I am a loner”), and another one, more labile, still marked by the selfhood of the person in time (i.e., “I am happy” or “I am a pensioner”), only the former being relatively preserved. Thus, AD patients, despite severe amnesia and cognitive disturbances, seem to keep a consistent, but impoverished, representation of themselves and stimulating their autobiographical memory seems relevant to support their self (El Haj et al., 2019; Rathbone et al., 2019).

Reminiscence workshops are classically proposed for AD patients in care units. Inspired by reminiscence therapy approaches, their principle is to improve psychological well-being using life histories (written, oral, or both). A review of the literature shows that reminiscence therapy benefits AD patients by improving cognition, depression status, and quality of life (Cuevas et al., 2020). Moreover, several weeks of reminiscence program can enhance subsequent autobiographical memory performances, including a significantly higher degree of episodic details (Kirk et al., 2019). However, these benefits are mainly observed for AD patients at the earlier stages of the disease, considering that such remembering activity is no longer achievable and beneficial for very amnesic patients at severe stages of the pathology.

Yet, AD patients at the severe stage of the disease maintain their sensory and emotional levels of musical appreciation, while other essential cognitive abilities (mnesic and verbal) are deteriorated (Norberg et al., 2003). Music indeed reduces anxiety and promotes social interactions (Guetin et al., 2013) and memories for musical material are especially resistant to AD (Groussard et al., 2019; Platel and Groussard, 2020). The impact of music workshops for AD patients in terms of emotional state has further been proved in other studies (Samson et al., 2009). In addition, the literature shows that recall of personal memories in AD patients can be facilitated by music stimulation (El Haj et al., 2012a,b; Palisson et al., 2015), that the complexity and richness of language production is enhanced when they listen to music (El Haj et al., 2013), and that music engages and potentially stimulates multiple aspects of the self in dementia (Baird and Thompson, 2018).

In this context we chose to study, in AD patients at moderate to severe stages of the disease, if repeated reminiscence workshops could induce an increase in the number and episodic quality of autobiographical memories, and the possibility that these repeated reminiscence workshops produced a long-term enrichment of their self-representations and their sense of identity. In a first and main study, we used excerpts of familiar songs as autobiographical memory inductors, and in a complementary study we proposed unfamiliar movie extracts as prompts. The purposes of the main study were (1) to examine if the repetition of musical reminiscence workshops had a positive effect on the recall of autobiographical memories, and if produced autobiographical memories following the sessions were more numerous and detailed. (2) if the assessment of the sense of identity of AD patients after these repeated workshops of musical reminiscence would show an enrichment of the self, corresponding to more details and positive description of themselves. The aim of the complementary study was to specifically verify whether we replicated the same results by changing the primer material presented in the reminiscence workshops, in order to try to disentangle the music and the repetition beneficial effects, respectively.

## Materials and Methods

### Participants

#### AD Patients and Matched Control (MC) Participants

We included a group of 20 patients diagnosed with probable AD according to the NINCDS–ADRDA criteria (McKhann et al., 1984), aged 76–92 years residing in nursing homes. All these residents have major cognitive impairment (MMSE between 4 and 15, *m* = 8.9). A group of 20 Matched Controls (MC) was constituted from the residents living in the same nursing homes. We measured patients' and MCs' cognitive efficiency with Signoret's cognitive battery (BEC96; Signoret, 1988), which includes several subtests assessing recall, learning, orientation, mental manipulation, mental problem-solving, verbal fluency, object-naming, and visual reproduction (maximum score: 96), which is roughly similar to the MMSE, but with a greater sensibility and validation with French speaking populations. Depression was controlled with the Geriatric Depression Scale (GDS) (Yesavage et al., 1983), and autobiographical memory was assessed with a semi-structured questionnaire, the “TEMPau” (Piolino et al., 2000) in a simplified version with three periods of life: childhood and teenage period (0–17 years), young adulthood (17–30 years), and the period after 30 years old. For each time period, participants were required to produce detailed memories of specific personal events and to state when and where each event occurred. They were instructed to describe personal events with as much details as they could and each event had to belong to one of the four topics: an event related to a person, a school or a professional event, a trip or a journey, or a family event. If participants were unable to recollect spontaneously a specific event, they were encouraged to be specific if their memory regarded an event that was repeated or extended in time). Each event was scored on a 4-point episodic scale, which takes into account the specificity of the content (single or repeated event), spatio-temporal situation, and the presence of details (perceptions, thoughts, feelings…).

A statistical comparison of these groups using Welch's Independent Samples *T*-Test (accounting for unequal samples variances) showed that our two groups were not different in age and socio-cultural level, and that their depression scores were not statistically different either. There was an expected significant difference between AD and MC regarding their performances on the MMSE, BEC96 and TEMPau tests (see Table 1).

**Table 1:** Description and comparison of the sample populations.

	**AD**	**MC**	***t***	***p***
	**Mean/SD**	**Mean/SD**		
Age	84.2/6	82/10	0.861	0.396
Education	6.3/2	5.5/2	1.275	0.210
GDS	4.2/2.6	3/2.4	1.506	0.140
MMSE^*^	11.5/4.3	26.7/2.3	−14.033	< .001
TEMPau^*^	4.3/1.7	8.2/1.4	−8.012	< .001
BEC 96 Total^*^	36.7/18.6	81.5/7.4	−9.989	< .001

* *Significant differences, Welch's Independent Samples T-Test*.

*BEC 96 (Signoret, 1988)*.

*GDS, Geriatric Depression Scale (Yesavage et al., 1983)*.

*TEMPau, Autobiographical memory assessment task (Piolino et al., 2000)*.

*AD, Alzheimer disease; MC, Matched Controls*.

#### Experimental Procedure

**Figure 1:**
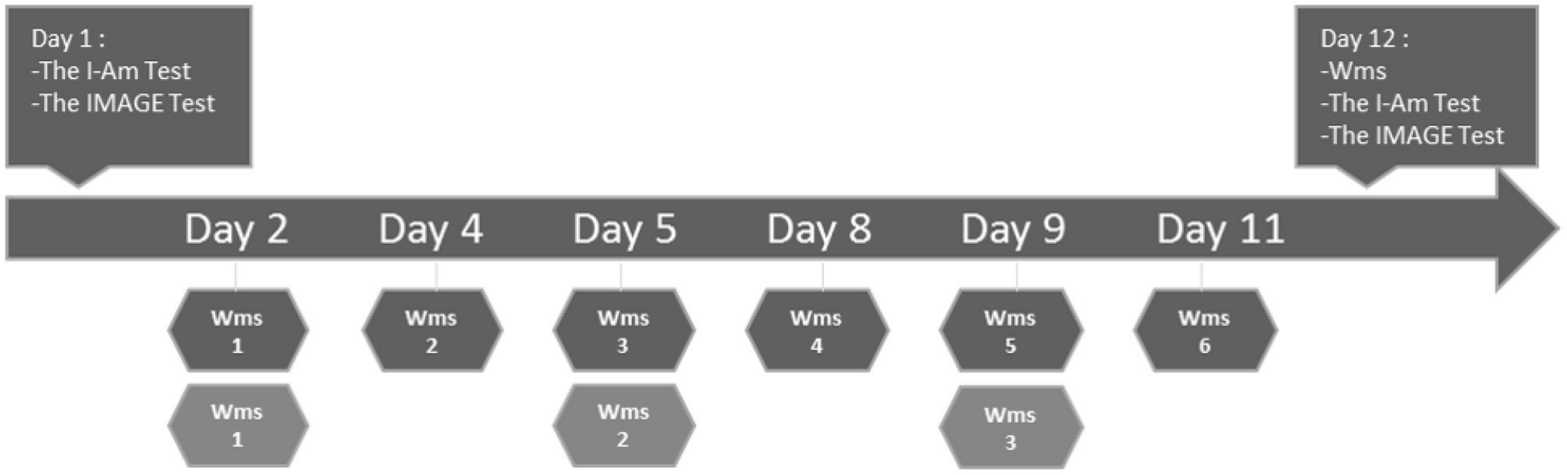
Illustration of the experimental procedure. Wms, Workshops of memories reminiscence.

This procedure was organized on a 12-day period, Monday (D1), Tuesday (D2), Thursday (D3) and Friday (D4) for the first week and with the same days (8–12) for the second week (Figure 1). The workshop of reminiscence activities was part of daily routine set by neuropsychologists in training. This study respected the Declaration of Helsinki and consent was asked before the beginning of the study from the family and participant and was systematically asked again before each session. All workshops took place in the same way and in the same location. A room was prepared in advance by the examiner who used a music diffuser to broadcast music and a voice recorder to record all sessions, allowing the use of these records to score memories. On Day 1, an individual assessment was performed on the sense of identity with the IMAGE and the I-AM Tests (see hereafter for the detailed description of these tests). On Day 2, participants joined groups of musical reminiscence workshops using three popular songs as cues to promote autobiographical memory retrieval. The AD group attended six sessions (Day 2 to Day 11). Considering that the MC participants could remember perfectly well their involvement to a previous workshop, we proposed that they participate only to three workshops (Day 2, Day 5, and Day 9), hence avoiding boredom and premature withdrawal from the experiment. During each workshop session, we counted the number of interactions between participants, and the number of memories and probed memories produced. On day 12, an individual assessment of autobiographical memory took place by proposing to listen to the cues from songs used during the reminiscence workshops. We also re-assessed on day 12 the sense of identity (IMAGE and the I-AM Tests) for all the participants.

#### Sense of Identity Assessment (Day 1 and Day 12)

##### The IMAGE Test

The IMAGE test was adapted for use with AD patients in the advanced stage of dementia, from the second edition of the Tennessee Self-Concept Scale (TSCS2, Fitts and Warren, 1996), the Questionnaire of Self-Representations (QSR; Duval et al., 2012), and the revised version of the Self-Consciousness Scale (Scheier and Carver, 1985). The IMAGE test is composed of 24 descriptive statements. All the sentences were worded so that they would be easily understood by AD patients and pretests of comprehension were made in a different sample of patients. This test was previously used and validated in Eustache et al. (2013). The examiner presented each descriptive statement in the form of a question to the participant who was asked to answer whether it was true or false. The examiner then asked the participant to say whether the statement was totally true/false or only partly so. Instructions were repeated when necessary. Responses were recorded by the examiner and assessed on a 4-point Likert-like scale with the following anchor points: totally false (1), partly false (2), partly true (3) and totally true (4). The scoring procedure was adapted to take the general meaning of the sentences into account.

##### The I-AM Test

The I-AM test is an adaptation of the Twenty *Statements Test* made for the Eustache et al. (2013) study, which requires participants to complete 10 written sentences beginning with “I am.” The test takes the form of a private discussion with the examiner, in which the participants give 10 descriptions of themselves. The examiner writes down the participants' answers and, if necessary, encourages them to continue in as neutral way as possible. No time limit is imposed, but in practice the test lasted <20 min.

Each response is classified as idiocentric sentence, small group, large group, or allocentric sentence. Idiocentric responses reflect the independent type of self-construal, that is, statements refer to personal qualities, attitudes and states that are not related to other people. By contrast, small-group and large-group statements reflect interdependent construal of self. Allocentric responses are socially oriented by nature, as they involve interdependence and other people's viewpoints. Finally, if applicable, we noted the emotional valence associated with each statement. A double quotation is done for each statement provided by participants, until a consensus is reached.

The following scores were calculated for each session: the overall number of statements generated, the number of responses belonging to each category or subcategory, and their emotional valence (positive, negative, or neutral,). We also counted the number of sentences in which participants clearly stated that they no longer knew who they were, and sentences that were obviously erroneous at the time of testing (e.g., “I am a student”), but which could have been true in the past.

#### Reminiscence Workshops With Song Extracts as Primers

We proposed “musical” reminiscence workshops to all participants. These workshops included 4 or 5 participants. We chose three famous songs from the repertoire of French variety. These songs refer to specific thematic or special events that can serve as relevant cues for recovery. We selected “*Sacré Charlemagne*” (Holy Charlemagne) by France Gall (1964, evoking the period of childhood and school); “*Le petit bal perdu*” (The small lost ball) by Bourvil (1961, evoking the period of young adulthood and ballroom dancing); and “*La mer*” (The sea) by Charles Trenet (1946, evoking the period of adulthood and the holidays). The choice of songs for our study was guided by different criteria. These songs had to be famous and heard mostly during the period of reminiscence peak in AD patients. They were chosen, first, because they refer to strong topics in everyone's life and also because they are familiar enough to generate positive emotions and facilitate the reemergence of old memories. The familiarity of the songs was checked for all participants before their inclusion in the study.

After each listening, we asked every participant if they knew the song (notably to verify their level of familiarity with the song) and asked if this particular song triggered a personal memory. If memories were not spontaneously produced, we proposed different questions and assertions (see Appendix) to probe the reminiscence of episodic autobiographical events. We also reported the number of such incitations for each participant.

#### Scoring Autobiographical Memories During Workshops

All the memories produced during the workshops were transcribed verbatim and all memories produced by each participant was scored for episodicity using an evaluation grid inspired by the TEMPau test (cf supra). The maximum possible score was twelve points (see Table 2). One point was given for each of these criteria: the uniqueness of the event, the duration (<24 h), a general and a precise time and spatial localization. The richness of details and the emotional details were quoted on three points each: one point stands for one detail, two points for two, and three points for three or more details. We rated each memory for each song theme and calculated an average score of memory quality for each session and for each participant. Memories were scored independently by three different neuropsychologists (each one following specifically some of the participants, with coordination meetings to avoid a single quotator bias).

**Table 2:** Episodic assessment of autobiographical memories.

	**Single event**	**Duration <24 h**	**Time general**	**Space general**	**Temporal localization**	**Spatial localization**	**Richness of details**	**Emotional details**	**Total**
Quotation	1 point	1 point	1 point	1 point	1 point	1 point	0–3 points	0–3 points	/12

We also rated the global number of memories (beyond the episodic quality) produced by each participant at the workshops. For each workshop session, we added the number of memories produced for the three songs by each participant, and then calculated a mean number of memories by session for each group. Since the second session, we also noted the number of memories, which were identical to those produced during the previous session. In the statistical analysis, we took into account the number of same memories produced in the last two workshops. In order to evaluate the quality of communication and the dynamic effect of the groups, we rated the number of interactions between two or more participants (quoted during the workshops and verified with the audio records), and calculated an average score per resident and per session.

#### Hypotheses

Considering the different assessments of autobiographical memories and evaluation of the sense of identity before and after the reminiscence workshops, we made several assumptions: (1) we hypothesized that the number of autobiographical memories produced during the reminiscence workshops would significantly rise over sessions for both groups; (2) among the workshop sessions, we also expected a significant increase of the episodic quality of autobiographical memories (difference between the first and last workshops), particularly for AD patients; (3) we predicted a significant intensification of the number of communication exchanges between the participants between the first and last workshop sessions; (4) regarding the assessment of the sense of identity, we expected to confirm the stability of response profiles for the two groups for the IMAGE Test between the two assessments (Day 1 and Day 12), as found in Eustache et al. (2013); (5) for the I-AM Test, we predicted a significant greater number of allocentric sentences on Day 12 compared to Day 1; (6) knowing that the literature on reminiscence workshops shows a benefit of this support on self-esteem, we hypothesized that, for the I-AM Test, both groups would produce more positive sentences to describe themselves during the second evaluation.

##### Statistical Analyses

Considering the non-normality and the categorical nature of most of the data, all the statistical analyses were performed with non-parametric tests. Global progression of performances during the workshops was analyzed with a Friedman ANOVA. A Mann-Whitney *U*-test was used in order to compare the performances between groups for the different variables (number of exchanges, number of memories and number of incited memories), and a Wilcoxon test to compare the performances of each group between sessions. For the sense of identity (I-AM and IMAGE tasks), Wilcoxon and Khi2 tests were calculated to compare the profile of answers of each group between the two individual assessment. For Mann-Whitney and Wilcoxon statistical comparisons, significant results are provided with effect size: *d* = 0.2 can be considered a small effect size, *d* = 0.5 represents a medium effect size and *d* = 0.8 refers to a large effect size (Cohen, 1988). All the statistical results were considered as significant at *p* < 0.05.

## Results

### Autobiographical Memory

#### Global Changes of Performances During the Workshops Within Each Group

For the MC group we did not obtain any significant differences (Friedman ANOVA) between the three workshops of reminiscence regarding the number of autobiographical memories produced, the number of incentives provided by the neuropsychologist and the number of interactions between participants.

Conversely, regarding AD participants, we observed a significant increase of the number of memories (Friedman ANOVA, X2 = 25.55; *p* < 001) and of the quality of the memories throughout the six workshops (Friedman ANOVA, X2 = 42.64; *p* < 001). The average score for each topic songs also showed a significant progression (school: X2 = 22.88; *p* < 001; ball: X2 = 17.57; *p* < 001; sea: X2 = 33.93; *p* < 001). A comparison of the average episodic scores obtained for the six workshops did not show any significant difference between the three songs used. The same test showed a significant increase of the mean number of autobiographical memories produced, and a significant reduction of the mean number of probes provided by the neuropsychologist. The analysis showed a significant increase of the same memories produced (X2 = 30.74; *p* < 001). Concerning the communication during the workshops, the ANOVA of Friedman highlighted a significant increase of the mean number of exchanges between AD participants throughout the workshops (X2 = 19.96; *p* < 001).

#### Inter-Group Comparisons Focused on the First and Last Workshops

**Figure 2:**
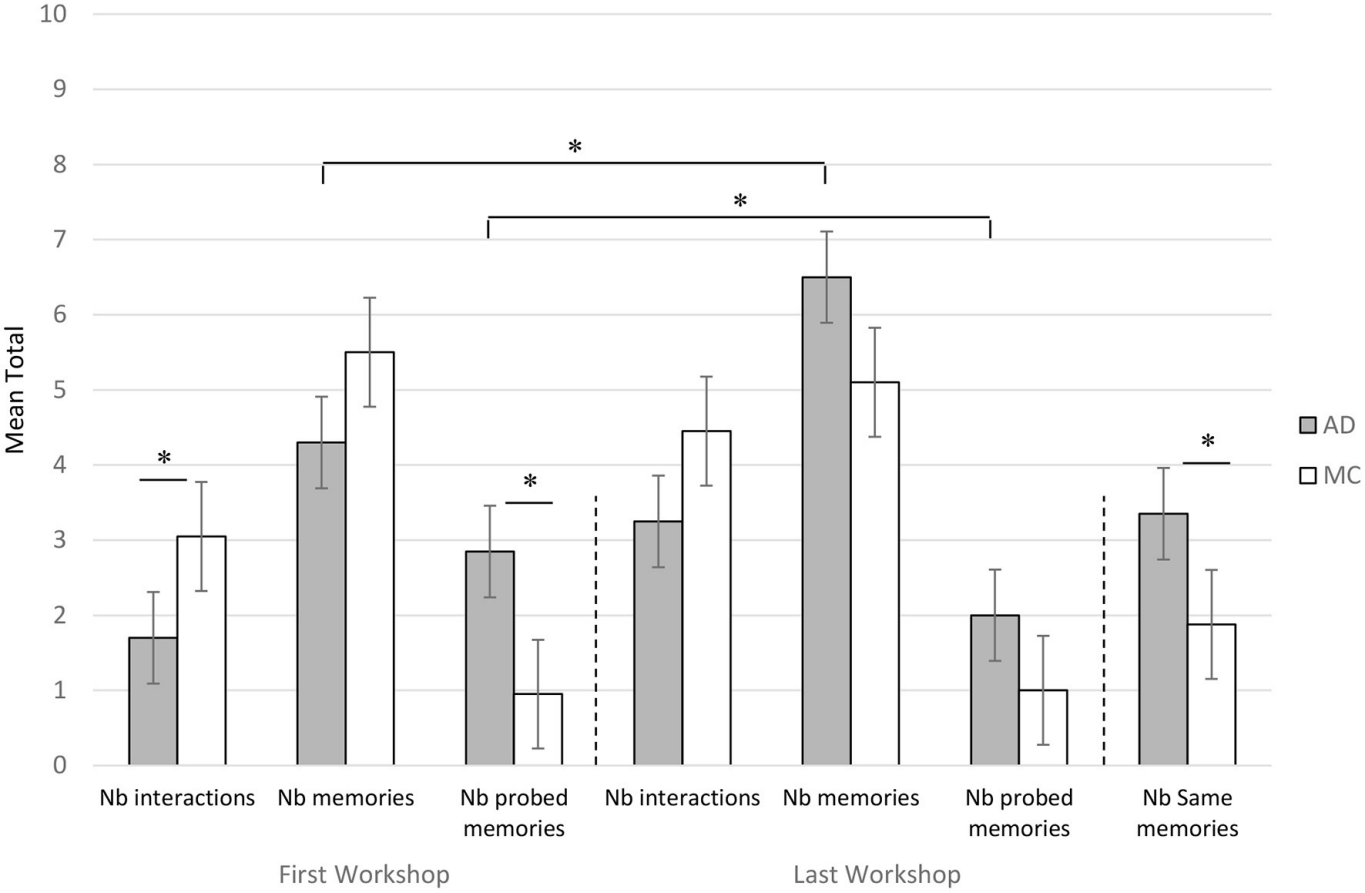
Average number of interactions, memories and probed memories produced by patients with Alzheimer's disease (AD) and Matched Controls (MC) for the first (FW) and last (LW) workshop sessions. Nb of same memories were counted on the basis of the last and the preceding workshops. *significant difference at *p* < 0.05.

Figure 2 shows the number of interactions, number of memories produced and number of probed memories at both times (first and last sessions), for each group. For the first reminiscence workshop, the number of interactions (higher for controls) and the number of probed (higher for AD) were significantly different between the two groups (Mann-Whitney, *z* = −2.24, Cohen's *d* = 0.527 and *z* = 2.57, Cohen's *d* = 0.971, *p* < 0.05). Conversely, the number of memories produced during this first workshop was not significantly different between AD and MC groups.

For the last reminiscence workshop, except for the number of memories produced (in favor of the patients, *z* = 2.16, Cohen's *d* = 0.681, *p* < 0.05), no significant difference was found between the two groups either for the number of interactions or for the number of probed memories (Figure 2).

#### Comparisons Between First and Last Workshops Within Each Group

In the AD patients' group, the number of memories significantly increased and the number of probed memories decreased between the first and last workshops (Wilcoxon Test, *z* = 3.44, Cohen's *d* = 1.091, *z* = 2.20, Cohen's *d* = 0.476, *p* < 0.05). None of the other comparisons were significant (Figure 2).

Regarding the MC group, the number of produced memories remained stable over the workshops (a light decrease is even observed).

Finally, the mean number of identical memories (same memories produced between the last two workshops) showed that the AD group produced significantly more identical memories (Mann-Whitney, *z* = 3.09, Cohen's *d* = 1.114, *p* < 0.05) that the MC group (Figure 2).

### Changes in Episodicity of the Produced Autobiographical Memories

#### Inter-Group Comparisons Focused on the First and Last Workshops

For the first session, the quality of the produced memories was significantly different between AD patients and MC participants (higher for the controls, *z* = −4.66, Cohen's d = 2.117, *p* < 0.05), while for the last session, it was no longer significant, with a comparable quality of memories produced by both groups.

#### Comparisons Between First and Last Workshops Within Each Group

**Figure 3:**
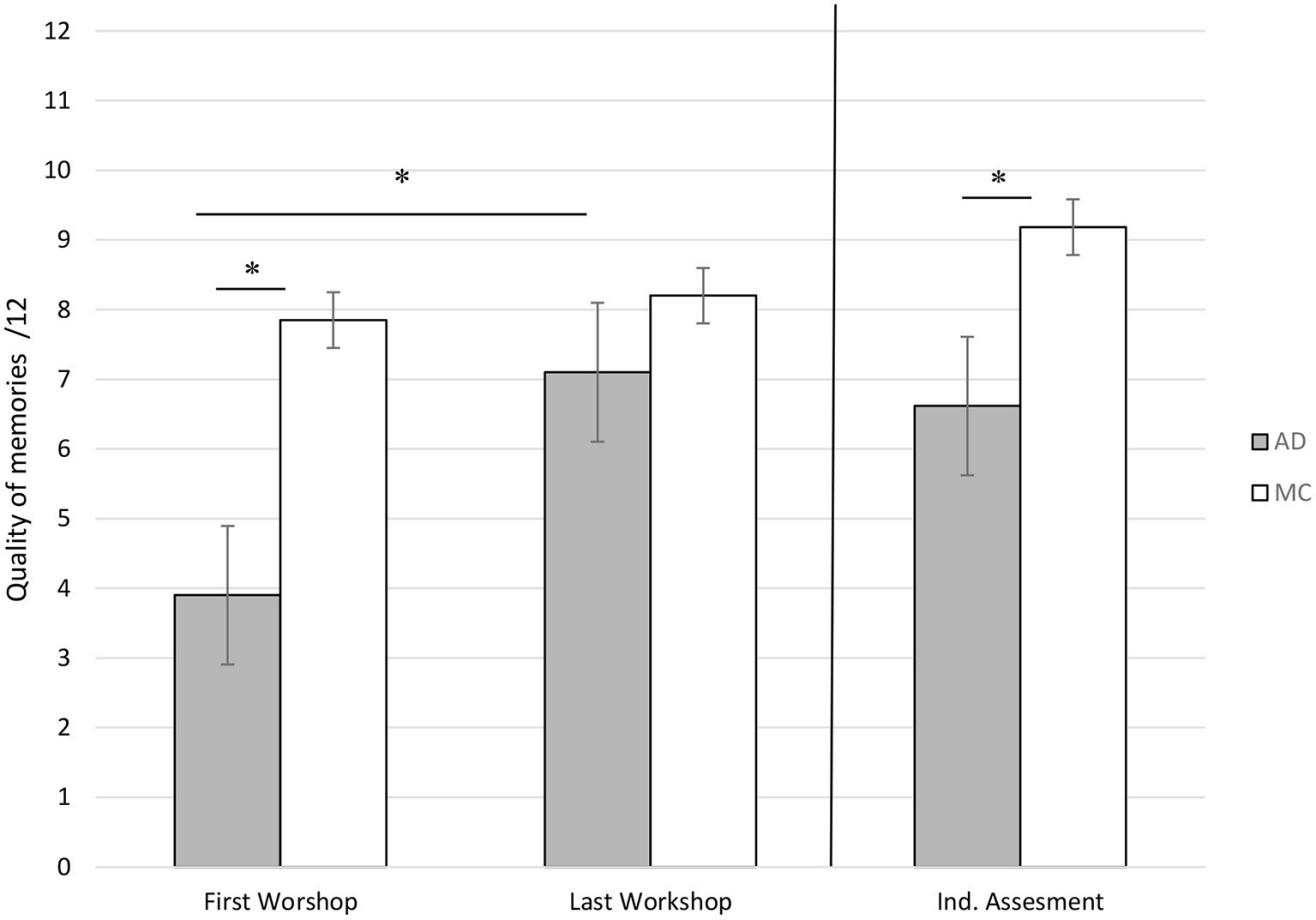
Mean scores of episodicity for memories produced by patients with Alzheimer's Disease (AD) and Matched Controls during the first workshop, the last workshop and the individual assessment. *significant difference at *p* < 0.05.

Among AD patients, in accordance with our second hypothesis, we showed a significant increase of the quality of memories (*z* = 3.82, Cohen's *d* = 1.912, *p* < 0.05), whereas the episodicity of the memories for MC participants remained stable between the first and last reminiscence workshops (Figure 3). This significant increase in memory episodicity in AD patients was observed independently for the three cue songs (school, ball, sea: *p* < 0.05).

#### Individual Assessment of Autobiographical Memory

Regarding the individual assessment of autobiographical memory (after the last reminiscence workshop), we found a significant difference for the episodic quality of memories between both groups (Mann-Whitney, *z* = −3.98, Cohen's *d* = 1.583, *p* < 0.05). The episodicity of the memories, which was significantly more detailed for MC at the time of the first assessment of autobiographical memory (before the workshops; Table 1), thus remained significantly better for the MC participants during this second individual assessment with the workshop procedure (Figure 3).

### The Sense of Identity

For AD participants, the statistical analysis (Wilcoxon test) comparing the individual distribution of the answers of each patient for the IMAGE test between the two individual assessments revealed that only three patients (out of the twenty) significantly modified the distribution of their answers, whereas only one participant in the MC group changed significantly her profile of answers. Overall, there were no significant differences between the mean global profiles of answers for both groups.

The qualitative analysis of the answers of the three patients showed that: (1) the first patient did not change his point of view between the two evaluations but used more extreme answers during the second evaluation. (2) the second patient changed six times the direction of his answers on the twenty-four items. (3) the third patient changed five times the direction of his answers on the 24 items. Concerning the participant of the MC group whose profile of answers changed significantly, he behaved like the first patient, with more extreme answers during the second evaluation, but going in the same direction. The qualitative examination of the frequencies of each answer among the two groups showed that, generally, the MC participants used more extreme answers. The statistical analysis (Khi2) did not exhibit any significant differences for the total distribution of answers on the IMAGE test between the two times of assessment, neither between the two groups of participants, which confirmed our fourth assumption.

Concerning the I-AM test, we did not confirm our assumptions (hypotheses 5 and 6). The statistical analysis (Wilcoxon test) did not show any significant difference for the AD group regarding the number of allocentric sentences. Concerning the number of sentences evoking the knowledge of a present state (which would show an updated self), there were no significant differences between the two evaluations. Moreover, the two groups did not produce more positive sentences to describe themselves during the second evaluation. Therefore, the content of the answers produced by the AD participants for the two sessions of the I-AM test stayed close (i.e., remained stable). The only significant difference between our two groups relied on the number of repetitions (same sentences) between these two individual assessments, which were significantly more numerous for AD patients (Wilcoxon test, *z* = 3.29, *p* < 0.05).

## Discussion

### Autobiographical Memory

The main objective of this work was to study the relevance of repeated reminiscence workshops with musical cues on the recall of autobiographical memories in AD patients at moderate to severe stages of illness. In our first hypothesis, we expected to find an increase in the number of autobiographical memories provided by both groups during the sessions. The statistical analyses confirmed our assumption only for AD patients while MC participants did not show any increase in the number of autobiographical memories produced. First, we observed a spectacular increase of the number of memories produced by the AD participants, considering their massive amnesia (no patient recalled taking part in the workshops), while MC participants produced less memories at the end of the workshop sessions. In fact, the stagnation of the number of evoked memories produced by the control participants is rather easy to explain. Indeed, since the second workshop session, the healthy participants remembered their previous participation, and tried not to produce the same memories as the day before. For the third session, we observed that most of the MC participants began to get bored by the exact same activity, some of them not being motivated anymore to produce personal events based on the songs. This result justifies that more sessions were not proposed afterwards to participants of the control group. On the contrary, AD patients fully benefited from the repetition of the workshops, producing more numerous and detailed autobiographical memories during the last workshops, exceeding our expectancies. Although this result appears quite impressive, the individual assessments of autobiographical memory, undertaken the next day after the last workshop, showed that patients still exhibited autobiographical memory impairment, in accordance with their performance on the TEMPau task during the first autobiographical assessment. Despite an obvious improvement of the episodic quality of the produced memories during the workshops individually, their memories stayed significantly less detailed than those of the MC participants and highlighted the predicted semantization of memories typically observed in AD patients (Piolino et al., 2009). During workshops, AD patients repeated the same memories significantly more often than MC participants (Figure 2), confirming a form of impoverishment of the repertoire of autobiographical memories. Moreover, we found that AD patients showed a significant decrease in the need to be helped during the workshops.

Additionally, we expected to find an increase in the number of interactions during the workshops. We indeed observed a significant increase of patients' interactions, contrary to MC participants, showing that AD patients felt better over sessions and improved their communication capabilities (El Haj et al., 2013). Here again, we show the positive impact of the repetition of these workshops, despite the lack of explicit memories of the sessions. These results are important in terms of care: they stress out the benefit of reminiscence workshops on the ability for residents to evoke personal memories, especially emphasizing a quality of memories that has improved enough to be compared to the one of MCs in the last session, thanks to the repetition (six sessions over 2 weeks) of the reminiscence workshops with the same musical cues.

### Sense of Identity

According to our fourth assumption regarding the “IMAGE” Test, we expected to replicate the stable profile of the answers between the first and second individual assessments carried out before and after the reminiscence workshops for each participant of this study (Eustache et al., 2013). Group analyses definitely showed a stability of the profile of the answers. Case-by-case analysis showed that only three out of 20 patients with AD, and only one participant from the MC group, significantly modified their answers between these two testing sessions. Qualitative analysis of the answers produced by these participants indicates that they do not vary in their valence (agreement vs. not agreement) but rather in their moderation, with AD patients producing more “extreme” answers (don't agree at all vs. don't agree that much; complete agreement vs. little agreement). Taken together it seems that the patients with AD exhibit the same type of performance as the control group when it comes to the stability of their answers between the Day 1 and Day 12.

Regarding the I-AM test, we first expected to underline an enrichment of the sense of identity on Day 12, resulting in the use of allocentric answers into greater number. We did not highlight this phenomenon to a significant degree. We therefore noticed that qualitatively, MC participants described themselves expressing the same ideas on D1 and D12, as the AD patients did, thus showing a great stability of the representation that they have of themselves. During workshops we noticed that the way patients referred to themselves was progressively more detailed, and we observed how pleased they were being solicited and sharing their personal memories. Nevertheless, we failed to objectivate a significant difference regarding the sense of identity in terms of details produced between D1 and D12. Similarly, the number of allocentric sentences or the number of sentences evoking the same idea, but formulated more positively, did not increase significantly with workshop sessions in the AD group. These observations go against our starting assumptions. One cannot exclude that this result may be due to the lack of sensitivity of our measurements of the sense of identity. Consequently, it would be interesting to develop a different methodological approach in order to obtain a more sensitive evaluation of the sense of identity, not in terms of coherence but rather in terms of richness of the self-representations. To overcome the lack of sensitivity of the selected scales measuring the sense of identity, finer content analyzes could have been considered, both for the production of sentences in the I-AM test and the production of autobiographical memories during workshops. In particular, it could have been interesting to use the scoring procedure of Singer et al. (Singer and Blagov, 2002; Blagov and Singer, 2004) in order to analyze whether the content of the autobiographical memories specifically depicted the patient personality traits. However, in the procedure proposed by Blagov and Singer (2004), participants were explicitly asked (usually in individual sessions) to produce autobiographical memories that describe themselves: « Participants were asked to generate 10 self-defining memories and then go back and rate each one of them on 12 emotions, vividness, and importance » which was neither the instruction nor the context of the workshops, which took place in small groups. Thus, it would undoubtedly be relevant to perform this kind of analysis only for individual sessions, carried out after the reminiscence sessions. Despite the positive impact of repeated reminiscence workshops on the number and quality of autobiographical memories, we failed to show a long-term positive effect on the sense of identity in AD. Thus, in spite of the obvious dynamic links between autobiographical memory and the Self as proposed by Conway (2005), the stimulation of autobiographical memory does not seem to be able to enrich the sense of identity in AD patients at moderate to severe stages of the illness. Effectively, mean MMS values of our AD participants are <12, while most studies targeted AD patients at an earlier stage of the disease, with a mean value of MMS >20 (Ben Malek et al., 2018; El Haj et al., 2019; El Haj and Allain, 2020). This does not mean that a “hard core” of identity is not preserved and stable in these patients, as proposed in some studies (Rathbone et al., 2019). More generally, the question of the identity in AD in the literature reveals contradictory results. Hence, the question remains of understanding whether the capability of updating their sense of identity is really impaired in AD patients. It may be that the number of reminiscence sessions was not sufficient in this work. It is also important to indicate that in most studies showing an enrichment of sense of identity following the production of personal memories, the evaluation of the identity is carried out immediately after the stimulation of the autobiographical memory (El Haj et al., 2019), while our experimental paradigm proposes a long-term measure of the sense of identity, 24 h after the completion of the last workshop, which constitutes certainly a delay which is too long to show any enriching effect in AD patients at this stage of the disease.

Further studies are needed in order to judge if the use of musical cues or the social emulation due to group interactions could positively modulate the sense of identity in moderate to severe AD patients. We could also suggest, as El Haj et al. (2015), that the songs must be chosen by the patients, as the authors exhibit that their AD participants produced more self-defining memories during exposure to their own-chosen music than to researcher- chosen music or during silence.

The analysis of the behavior of AD patients over the reminiscence sessions revealed an improvement of interactions. Beyond these observations, we observed a real pleasure taken by each patient during these workshops. In addition to increasing the number of social interactions, we noticed an increase in the use of humor and connivance between participants. One interesting point about our study is that MC participants were recruited in the same nursing home as AD patients (living in a specific Alzheimer Unit) and that the workshops were performed within the same institutions. As a result, we observed moments of sharing and curiosity for both groups in which everyone was able to share original stories about their lives. Residents were asked to discuss various topics and were aware of the experiences they shared, producing a group support. This daily work on themselves and other personal memories also seems to have a beneficial impact on interpersonal relationships outside the workshops, as reported by caregivers particularly about AD patients. All these clinical observations deserve to be quantified and objectified in future studies.

Even if the results concerning the enrichment of the sense of identity are rather disappointing, the impact of repetition of the reminiscence workshops on the increase in the number and the quality of autobiographical memories in very amnesic AD patients is particularly spectacular, and even exceeded our expectations. Can the repetition effect alone explain this result? And to what extent this benefit is brought by music as a primer of autobiographical memories? Our hypothesis is that, beyond the familiar, motivating and appealing dimension of the song excerpts used as memory primers, the main boosting effect of autobiographical memory is obtained by the repetition of the same workshop situation, and that we should find the same results with different materials. In order to test this hypothesis and the impact of another type of material as a primer, we proposed a complementary study to two other groups of patients and control subjects based on the same experimental design, except that the seed material are unfamiliar movie clips.

## Complementary Study-Reminiscence Workshops With Movie Extracts as Primers

In this complementary study, eight new AD patients (aged 77–91; MMSE score between 7 and 23, mean = 12.5) and six new paired controls participated in exactly the same paradigm, but using three movie extracts (1 min each, with dialogs and no familiar music) as primers (see hereafter).

Participants were included on the same clinical criteria as in the previous study. However, they come from another retirement home because it did not seem relevant, at the risk of having many bias in the interpretation of the results, to recruit the same participants to perform the same experiment by simply changing the primers.

As for the preceding study, statistical comparison of these new groups, using Mann-Whitney U Test, showed that they were matched in age and socio-cultural level, and that their depression scale scores were not statistically different. There was an expected significant difference between AD and MC regarding their performances on the MMSE, BEC96 and TEMPau tests (see Table 3), except for the “Mental Manipulation” subtest of the BEC96, were the statistical scores between AD and MC showed no difference (this was also the subtest with the smallest difference in the previous study).

**Table 3:** Description and comparison of the sample populations (Complementary Study).

	**AD**	**MC**	***W***	***p***
	**Mean/SD**	**Mean/SD**		
Age	86.1/5.08	88.8/2.92	15.500	0.298
Education	2.3/1.06	2.66/1.21	20.500	0.675
GDS	3.25/3.15	2.16/0.75	22.500	0.894
MMSE^*^	12.5/5.15	25.8/1.9	0.500	0.003
TEMPau^*^	3.33/1.11	8.89/1.07	0.000	0.002
BEC 96 Total^*^	37.1/12.8	81.8/7.2	0.000	0.002

* *Significant differences, Mann-Whitney U-Test*.

*BEC 96 (Signoret, 1988)*.

*GDS, Geriatric Depression Scale (Yesavage et al., 1983)*.

*TEMPau, Autobiographical memory assessment task (Piolino et al., 2000)*.

*AD, Alzheimer disease; MC, Matched Controls*.

There was no significant difference, between the profiles of the participants of the two studies, except for the level of education, which was significantly lower among the participants of this complementary study. The experimental procedure was strictly the same between the two studies (number of workshops, measures of sense of identity and episodicity, etc.), except for the material used to elicit autobiographical memories.

### Selection of Movie Extracts as Primers

The films selected correspond to the same thematic used in the previous study: school, the sea (seaside holidays) and ballrooms. These themes will serve as relevant cues for autobiographical recovery. Unlike the familiar songs chosen in the previous study, we have deliberately chosen scarcely known movies to avoid inducing false memories corresponding of the events present in the film (i.e., an extract of The longest day movie could induce in a patient who has seen this film many times the story of an event that corresponds to what happens in the film but not to a personal memory). In fact, none of the participants succeeded in identifying one of the three movies from the proposed extract.

We therefore selected “Le temps des portes plumes / The time of penholders” (school theme), a movie released in 2006 and whose story takes place in 1956, “Les vacances du petit Nicolas/Little Nicolas' holidays” (seaside holiday theme), released in 2014 and whose story takes place in the 1960s and “Le bal / Le bal” (ball theme), released in 1983 whose story evokes 50 years of balls from the 1920's.

We then selected a representative excerpt from each film of about 1 min that essentially evokes the thematic already mentioned. The selected extracts correspond to exchanges between different characters of the film in a representative context (classroom, beach, and ballroom). Unfamiliar background music was present during these excerpts.

## Results–Complementary Study

### Autobiographical Memory

We obtained a same global significant increase of the number of memories and of their quality throughout the six workshops for AD participants.

**Figure 4:**
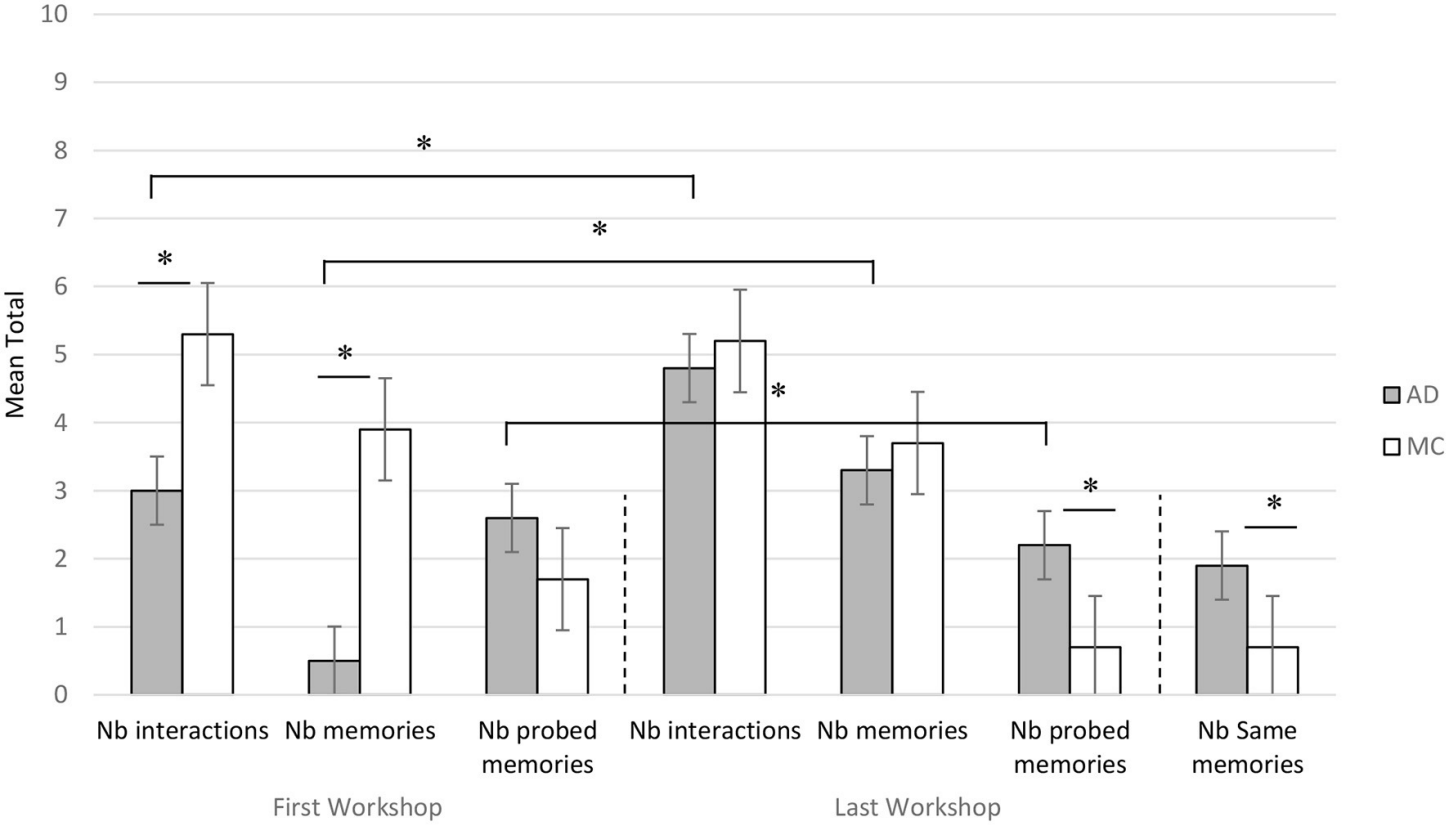
Complementary study: Average number of interactions, memories and probed memories produced by patients with Alzheimer's disease (AD) and Matched Controls (MC) for the first (FW) and last (LW) workshop sessions. Nb of same memories were counted on the basis of the last and the preceding workshops. *significant difference at *p* < 0.05.

More precisely, during the first workshop we showed that the number of memories as well as the number of interactions between participants were significantly different between the patients and the controls (higher for the controls, Mann-Whitney *z* = −3.08, Cohen's *d* = 3.546, *z* = −2.46, Cohen's *d* = 1.546, *p* < 0.05) as shown in Figure 4. Between the first and the last workshops, the number of memories as well as the number of interactions increased significantly for AD patients (Wilcoxon Test *z* = 2.03, Cohen's *d* = 2.307, *z* = 2.52, Cohen's *d* = 0.995, *p* < 0.05), while the results for the MC group were stable for these two variables. Only the number of probed memories decreased significantly for the MC group between the first and the last sessions (Wilcoxon Test *z* = 2.20, Cohen's *d* = 1.581, *p* < 0.05), while it remained stable for AD patients. These overall results were also found for each sub-theme (school, sea, ball). Moreover, the mean number of identical memories (same memories produced between the last two workshops) showed that the AD group produced significantly more identical memories (Mann-Whitney, *z* = 2.43, Cohen's *d* = 1.897, *p* < 0.05) than the MC group (Figure 4).

Comparing the results of this complementary study with the previous study, we obtained similar statistical results for the control groups, except for the number of interactions during the first workshop which was significantly more important for the participants of the MC group of the second study (Mann-Whitney, *z* = −2.16, *p* < 0.05). On the other hand, although the AD patients of this second study produced significantly more memories between the first and the last sessions, overall fewer memories than the patients from the first study were produced, both during the first and the last sessions (Mann-Whitney, *z* = 3.07, *p* < 0.05).

**Figure 5:**
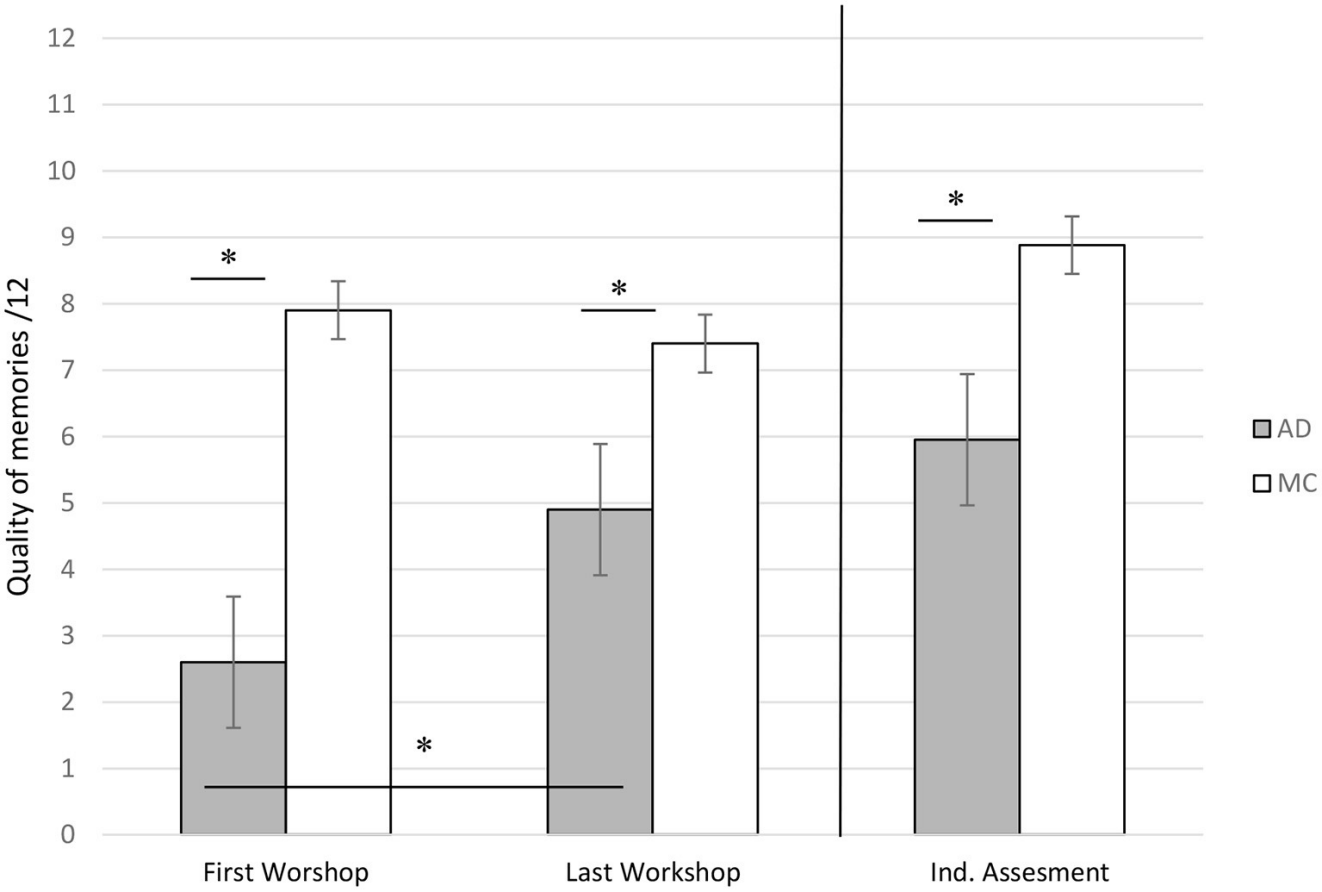
Complementary study: Mean scores of episodicity for memories produced by patients with Alzheimer's Disease (AD) and Matched Controls during the first workshop, the last workshop and the individual assessment. *significant difference at *p* < 0.05.

Regarding the quality of memories (episodic assessment, see Table 2), as in the previous study, we found a significant increase in the episodicity of memories for AD patients between the first and the last workshops (Figure 5). However, the difference remains significant between patients and controls during the last workshop, except for the theme of school where there were no statistical differences in the quality of the memories during the last session between AD patients and controls. Thus, we obtained similar results between the two studies although AD patients in the second study produced significantly less episodic memories during the last workshop (Mann-Whitney, *z* = −2.13, *p* < 0.05). Nevertheless, the evaluation of autobiographical memory in the TEMPau before the start of the workshops did not show any significant difference between the studies for the two groups of patients.

Regarding the results of the two tests measuring the sense of identity, as in the previous study no significant difference was revealed in the profiles of responses to the IMAGE test between the two individual assessments before and after the workshops. Likewise, the Wilcoxon test does not exhibit a significant difference for the I-AM test concerning the number of sentences produced, the positive and negative valences of the sentences, as well as the allocentric and idiocentric character of the productions between the two evaluation sessions for both patients and controls. They produce the same number of positive and negative valence sentences and the same number of allocentered and idiocentric output between the two sessions.

## Discussion–Complementary Study

While the results of this complementary study largely reproduce those already obtained in the previous one, they are not entirely superimposable. We very clearly confirm the stability of AD patients' responses for the two sense of identity tests proposed (IMAGE and I-AM), despite an advanced stage of Alzheimer's disease and a massive amnesia. This stability of identity is remarkable as it shows that memory loss in Alzheimer's disease does not completely abolish the sense of identity, contrary to popular belief (Eustache et al., 2013; Rathbone et al., 2019). Even if the patients describe themselves more poorly than the controls, they know very well who they are, and are able to describe their personality traits, confirming an undeniable integrity of the personal semantic memory marked by sameness of the person. On the other hand, as in the previous study, we showed no enrichment of identity following the repetition of reminiscence workshops. As we already proposed in the discussion of the main study, this result may be explained by the fact that our identity assessment tools are not sufficiently sensitive or adapted to the situation, or that the number of sessions may not be sufficient to achieve a long-term significant impact of these reminiscence workshops on self-enrichment.

The most spectacular result of these two studies is the increase in the number and quality of memories evoked by patients throughout the sessions. However, while using film clips as primers, the autobiographical memory productions of AD patients did not reach the same level as in the main study where patients produced almost as detailed memories during the last workshop than the control participants (see Figure 3). This difference can be explained by various factors, notably a much smaller sample of participants in the complementary study, and patients with a significantly lower level of education than the participants of the main study, and of course a possible lower impact of unfamiliar movie clips as primers of autobiographical memories. Therefore, we cannot fully conclude that this enrichment phenomenon of autobiographical memories is solely the consequence of the repetition of the sessions. The quality and attractiveness of the media used in the workshop session to prime autobiographical memories certainly generates an impact, as clearly shown in the literature on the power of music on patient behavior (Guetin et al., 2013; Platel and Groussard, 2020) and the specific resistance of the long-term musical memory in this pathology (Groussard et al., 2019). Moreover, the fact that in the first study the songs used are very well-known may produce a feeling of “déjà vu” and facilitate reminiscence with the support of emotion.

Still, even if the magnitude of the result of the second study did not equal the ones of the first, we again very clearly see a benefit of repetition during the sessions in patients. Although patients do not explicitly remember previous workshops, their personal memories, very poor and semantic during the first session, become more numerous, rich, and detailed. Thus, each previous session appears to leave some implicit trace that allows a more fluid evocation during the following sessions, and the neuropsychologists who led these workshops noted that, even if the patients say they do not remember them and the previous workshops, their attitude reveals a growing feeling of familiarity with the situation during the workshops. This effect corresponds to what we have documented elsewhere (Coppalle et al., 2019, 2020) concerning the increase in familiarity-based recognition following repeated exposure to various initially unknown information (music and paintings). The adaptation of the patients and the increase in memories produced during these reminiscence workshops would be mediated by an implicit encoding of the situation through the repetition effect.

## Conclusion

These results show a real and strong benefit from the activity of reminiscence on the evocation and quality of memories for AD residents, mainly attributable to the intensive repetition of these workshops (6 over 2 weeks), with autobiographical memory productions almost similar to those of controls at the end of the 2 weeks.

Regarding the sense of identity, we highlighted the stability of self-representations with both tests (IMAGE and I-AM), confirming previous results and a maintenance of a core representation of themselves in moderate to severe stages of AD patients. However, contrary to our hypotheses, we did not obtain a significant enrichment of the sense of identity, possibly due to a limitation of sensitivity of our measurement tools. Yet, professional caregivers and neuropsychologists perceived an improvement in self-esteem and relationships between residents.

Overall, the main message of our study is the clear-cut improvement of autobiographical memory retrieval capabilities even in the severe stage of AD, due to the steady repetition effect of the workshops. We must now evaluate the long-term durability of this improvement, and the differential and lasting impact of the material used as primers. Undoubtedly artistic medium like music, which convey cultural references and emotions, are good primers of personal event memories. These results also constitute a message of encouragement for professionals in nursing homes and families as they highlight preserved capabilities that could be shown through the daily care routine or workshops that they provide. Furthermore, these preserved learning capabilities can be used to improve the quality of life of AD patients and should encourage us to promote non-pharmacological interventions.

## Data Availability Statement

Raw data supporting the conclusions of this article can be provided by the authors upon request.

## Ethics Statement

Ethical review and approval was not required for the study on human participants in accordance with the local legislation and institutional requirements. The patients/participants provided their written informed consent to participate in this study.

## Author Contributions

HP, M-LE, AV, FE, and BD designed the paradigm and coordinated the study. HP and M-LE wrote the manuscript, supervised and contributed to the data collection. HP, RC, and MG analyzed the data. All authors proofread the manuscript.

## Conflict of Interest

The authors declare that the research was conducted in the absence of any commercial or financial relationships that could be construed as a potential conflict of interest.

## Appendix

Examples of solicitation questions to facilitate the retrieval of autobiographical memories in AD patients:

For each song, we formulated questions to grasp patients' attention and to help them remember what was asked. These questions served also to generate more details when memories were not spontaneously produced.

- For the first song, here are the types of questions that we have listed: “This song is about childhood. Can you give a memory of your childhood at school? Can you give me a memory from school when you were a child?

“Imagine being in your class at school, as a child: what are you doing?”

“Who did you like playing with? What game did you play at recess?”

“Remember the village where you went to school?≫”

“Did you like your teacher? ≫”

“Was it a good period for you? ≫”

“Do you recall good memories? Which ones?”

- For Song 2: “Can you tell me a childhood memory? Were you in the local dance troop? Did you like to dance?

“Where would you go dancing? Would you meet friends, boy/girl friends?”

“Was it a good period for you? Do you recall good memories? Which ones?”

- For Song 3: “Have you ever seen the sea? Do you like the sea? Did you like to go there? Tell me a memory at the sea?”

“Where did you like taking walks in your adult life? With your children? When they were young? Tell me a memory outdoors? ”

“Was it a good period for you? ≫

“Do you recall good memories? Which ones?”
